# ERN GENTURIS clinical practice guidelines for the diagnosis, treatment, management and surveillance of people with schwannomatosis

**DOI:** 10.1038/s41431-022-01086-x

**Published:** 2022-04-01

**Authors:** D. Gareth Evans, Stefania Mostaccioli, David Pang, Mary Fadzil O Connor, Melpo Pittara, Nicolas Champollion, Pierre Wolkenstein, Nick Thomas, Rosalie E. Ferner, Michel Kalamarides, Matthieu Peyre, Laura Papi, Eric Legius, Juan Luis Becerra, Andrew King, Chris Duff, Stavros Stivaros, Ignacio Blanco

**Affiliations:** 1 Manchester Centre for Genomic Medicine, Division of Evolution and Genomic Sciences, University of Manchester, MAHSC, St Mary’s Hospital, Manchester University Hospitals NHS Foundation Trust, Manchester, UK; 2 IDI-Istituto Dermopatico Immacolata Rome, Rome, Italy; 3 Italian Association for NF2 and Schwannomatosis Patients NF2 Project Aps, Rome, Italy; 4 Pain Department, Guy’s & St Thomas NHS Foundation Trust London, London, UK; 5 Schwannoma Support UK, London, UK; 6 NF Patients United (NFPU), Nicosia, Cyprus; 7 Dept of Dermatology, APHP, UPEC, Henri-Mondor Hospital, Créteil, France; 8 Department of Neurosurgery, King’s College Hospital London, London, UK; 9 Department of Neurology, Guy’s & St Thomas NHS Foundation Trust London, London, UK; 10 Department of Neurosurgery, Assistance Publique–Hôpitaux de Paris, Hôpital Pitié-Salpêtrière, Sorbonne Université, Paris, France; 11 Department of Experimental and Clinical, Medical Genetics Unit, Biomedical Sciences “Mario Serio”, University of Florence, Florence, Italy; 12 Department of Human Genetics, University of Leuven, KULeuven, Belgium; 13 University Hospital Leuven, Leuven, Belgium; 14 Neurology service, Neurosciences Department, Hospital Germans Trias I Pujol, Institut Català de la Salut, Barcelona, Spain; 15 Geoffrey Jefferson Brain Research Centre, Northern Care Alliance NHS Group, Manchester Academic Health Science Centre, University of Manchester, Manchester, UK; 16 Department of Plastic Surgery, Wythenshawe Hospital, Manchester University NHS Foundation Trust, Manchester, UK; 17 Clinical Genetics Department, Hospital Germans Trias I Pujol, Barcelona, Spain

**Keywords:** Cancer genomics, Whole body imaging

## Abstract

A Guideline Group (GG) was convened from multiple specialties and patients to develop the first comprehensive schwannomatosis guideline. The GG undertook thorough literature review and wrote recommendations for treatment and surveillance. A modified Delphi process was used to gain approval for recommendations which were further altered for maximal consensus. Schwannomatosis is a tumour predisposition syndrome leading to development of multiple benign nerve-sheath non-intra-cutaneous schwannomas that infrequently affect the vestibulocochlear nerves. Two definitive genes (*SMARCB1*/*LZTR1*) have been identified on chromosome 22q centromeric to *NF2* that cause schwannoma development by a 3-event, 4-hit mechanism leading to complete inactivation of each gene plus *NF2*. These genes together account for 70–85% of familial schwannomatosis and 30–40% of isolated cases in which there is considerable overlap with mosaic NF2. Craniospinal MRI is generally recommended from symptomatic diagnosis or from age 12–14 if molecularly confirmed in asymptomatic individuals whose relative has schwannomas. Whole-body MRI may also be deployed and can alternate with craniospinal MRI. Ultrasound scans are useful in limbs where typical pain is not associated with palpable lumps. Malignant-Peripheral-Nerve-Sheath-Tumour-MPNST should be suspected in anyone with rapidly growing tumours and/or functional loss especially with *SMARCB1-*related schwannomatosis. Pain (often intractable to medication) is the most frequent symptom. Surgical removal, the most effective treatment, must be balanced against potential loss of function of adjacent nerves. Assessment of patients’ psychosocial needs should be assessed annually as well as review of pain/pain medication. Genetic diagnosis and counselling should be guided ideally by both blood and tumour molecular testing.

## Introduction

Schwannomatosis is an inherited syndrome characterised by the development of typically painful, benign nerve-sheath tumours (schwannomas) on the spinal and peripheral nerves around the body [1, 2]. Cranial nerves are affected to a lesser extent and there is characteristic sparing of the 8th cranial nerve, which is the most commonly affected by schwannomas in sporadic/isolated non-hereditary cases and in neurofibromatosis 2 (NF2) [1, 2]. Intradermal schwannomas are characteristic lesions in NF2 and are absent in schwannomatosis. Vestibular schwannomas may occur in around 10% of *LZTR1*-related schwannomatosis patients but do not seem to occur at any increased frequency in other types of schwannomatosis.

The ‘term’ schwannomatosis appears to date from the 1950s, but other terms such as neurilemmomatosis have also been coined. The early literature is confused as both schwannomatosis and neurilemmomatosis were terms used in Japan to include patients who clearly had NF2 with bilateral vestibular schwannomas [3, 4]. Nevertheless, in the mid-1990s a consensus began to develop that the entity schwannomatosis was distinct from NF2 [5–7], although concern still existed over significant overlap with NF2 [8]. The molecular mechanism of schwannomatosis shows different somatic point mutations in *NF2* between schwannomas in the same person [9]; linkage analysis in a number of families to exclude the *NF2* locus on chromosome 22q [10] confirmed the existence of the separate entity. In 2007 a separate gene on chromosome 22 called *SMARCB1* was found to cause a subset of familial and sporadic/isolated cases of schwannomatosis [11–14]. The gene was also linked in at least some families to a tendency to develop meningiomas [15], although this tumour is still relatively uncommon even in *SMARCB1*-related schwannomatosis [2, 16]. Seven years after identification of *SMARCB1* as a causal entity, a second 22q gene *LZTR1* was identified as a cause of schwannomatosis [17]. This again raised the overlap with NF2 as a number of cases developed unilateral vestibular schwannoma and met the Manchester diagnostic criteria for NF2 [18–20]. Furthermore, many sporadically affected individuals that do not have either *LZTR1* or *SMARCB1* germline pathogenic variants but meet schwannomatosis criteria [21, 22], have mosaic NF2 with identical pathogenic variants in two separate schwannomas [2, 20, 23, 24]. The overlap from both the vestibular schwannomas occurring in LZTR1-related schwannomatosis and mosaic NF2 mimicking schwannomatosis has necessitated a re-evaluation of the existing diagnostic criteria [25] and an international effort has defined new criteria that will be published in 2022.

The overriding feature in individuals with schwannomatosis is pain, with little if any neurological deficit [1]. Removal of schwannomas often results in complete resolution of pain symptoms [1]. Life expectancy is not usually reduced, unlike in NF2 [2], but quality of life is strongly affected. Whilst there exists some concern over malignant potential in SMARCB1-related schwannomatosis [26, 27], this does not appear to be a feature of other types of schwannomatosis. Other common features of NF2 such as ependymomas and ocular features such as retinal hamartoma, epiretinal folds and juvenile cataracts have not been reported in schwannomatosis [1, 2]. Until now only a guideline for children and young adults has been published [28]. Overall, *SMARCB1*/*LZTR1* have been shown to account for 70–85% of familial schwannomatosis and 30–40% of isolated cases in which there is considerable overlap with mosaic NF2. It is likely that at least one other gene/mechanism exists to explain 22q related schwannomatosis as well as at least a minority of cases caused by a non 22q mechanism.

## Scope of the guideline

This guideline is intended to define the optimal diagnosis, clinical management and surveillance of people with a confirmed diagnosis of schwannomatosis and has been elaborated by members of the European Reference Network (ERN) for Genetic Tumour Risk Syndromes (GENTURIS).

It aims specifically to integrate available information to assist healthcare professionals in the identification and clinical management and surveillance of people with schwannomatosis. These guidelines do not signify nor intend to be a legal standard of care, they should support clinical decision making, but never replace clinical professionals.

## Methods

The ERN GENTURIS schwannomatosis Guideline Group (GG) consists of clinicians with expertise from clinical genetics, (neuro-, peripheral nerve) surgery, dermatology, anaesthesiology, neurology, radiology, and affected individuals and their representatives. The GG was led by a Core Working Group of ERN GENTURIS Healthcare Provider (HCP) Members from different Member States and who are recognised experts in specialised clinical practice in the diagnosis and management of schwannomatosis. A Patient Advisory Group was established and included 4 affected individuals that have experience with schwannomatosis.

The guideline was developed based on 237 published articles extracted from PubMed, using the following terms: schwannomatosis [title/abstract].

Additional papers were requested from experts in the field and references of all the papers were considered. Papers were included if they contained any data on diagnosis, treatment, management or surveillance of people with schwannomatosis.

As is typical for many rare diseases, the volume of peer-reviewed evidence available to consider for these guidelines was small and came from a limited number of articles, which typically reported on small samples or series. To balance the weight of both published evidence and quantify/wealth of expert experience and knowledge, we have used the following scale to grade the recommendation: (i) strong evidence: Expert consensus AND consistent evidence; (ii) moderate evidence: Expert consensus WITH inconsistent evidence AND/OR new evidence likely to support the recommendation, and (iii) weak evidence: Expert majority decision WITHOUT consistent evidence. Expert consensus (an opinion or position reached by a group as whole) or expert majority decision (an opinion or position reached by the majority of the group) is established after reviewing the results of the modified Delphi approach within the Core Working Group.

After drafting recommendations amongst the GG these were subjected to a modified Delphi assessment. Delphi is a structured communication technique or method in which opinions of a large number of experts are assessed on a topic in which there is no consensus, and this was used as a consensus building exercise. Experts included in this exercise included the members of the Core Working Group, the Schwannomatosis GG, the Patient Advisory Group, as well as other (external) experts identified by the GG.

The survey existed of four rounds, in which the threshold for consensus was defined by a simple majority of the survey participants agree with the recommendation (>60% rated ‘agree’ or ‘totally agree’). Recommendations were graded using a 4-point Likert scale (totally disagree, disagree, agree, totally agree) and a justification for the given rating was obligatory. Even if consensus was met recommendations were still modified if a higher consensus was thought achievable from written responses. The facilitator of the Delphi survey provided anonymised summaries of the experts’ decisions after each round as well as the reasons they provided for their judgements. The recommendations are presented in the Table 1.

## Discussion

The schwannomatosis GG has developed recommendations for the diagnosis, surveillance and treatment of schwannomatosis in both children and adults with a high degree of consensus across clinical experts and patients. Recommendations are similar to, but distinct from a previous working group for the American Association of Cancer Research [28] which made recommendations for children and young adults. These differences are partly based on subsequent publications, but also on the need to simplify the gene-based recommendations as the childhood onset differences between LZTR1-related schwannomatosis and SMARCB1-related schwannomatosis are not striking. The present guideline has developed more comprehensive recommendations for treatment especially of the hallmark symptom of pain. Recommendations regarding surveillance are tempered by the need not to overburden patients with unnecessary MRI scans particularly as some schwannomatosis patients are mildly affected and may produce only a very small number of symptomatic schwannomas in their lifetime. We have also recognised that in an era of increasing large gene panel testing, exome and genome screening that ‘incidental’ presumed pathogenic variants in particular in *LZTR1* will be identified in individuals with no family history or suggestive personal history of schwannomatosis. Overall, the frequency of presumed loss of function variants in *LZTR1* in the population database gnomAD is ~1 in 310. Whereas the frequency of confirmed LZTR1-related schwannomatosis based on a birth incidence of schwannomatosis of 1 in 69,000 and the fact that around 27–30% of schwannomatosis cases are caused by *LZTR1* [2] is less than 1 in 227,000, This means that <1% of those individuals with no family history or suggestive personal history of schwannomatosis and carrying a potential pathogenic *LZTR1* variant are likely to develop schwannomatosis. This contrasts with a 50% likelihood of inheriting a disease associated variant in offspring of people with schwannomatosis and a pathogenic variant in *LZTR1*. Nonetheless, several cases of incomplete penetrance have also been observed for this gene even within families with confirmed cases [17, 18, 20, 24, 29–31], although the penetrance is not yet determined it may be in the region of 40–50%. Penetrance appears higher in *SMARCB1* and Loss of Function variants are much less common in population databases. As such we have not recommended surveillance in individuals with ‘incidental’ findings of an *LZTR1* variant. Genetic counselling should be guided ideally by both blood and tumour molecular testing to aid discussion of transmission risks. This should also address uncertainties around disease penetrance as well as informing about reproductive options.

Malignancy is thought to occur rarely in schwannomatosis. Recently several cases have been described mainly in patients harbouring germline mutations in *SMARCB1* gene. A clear increased risk of a malignant-peripheral nerve-sheath tumour has been established [26] although it is possible that a more extended malignancy phenotype associated with a *SMARCB1* pathogenic variant does exist [27]. Due to this increased risk, we have recommended that a changing tumour, in someone with *SMARCB1* germline pathogenic variant, especially one causing functional impairment, should prompt exclusion of malignant transformation.

Clinically, schwannomatosis is distinguished from NF2 by the absence of bilateral vestibular schwannomas and ependymomas [2, 25]. Previously, a vestibular schwannoma was considered an exclusion criterion for schwannomatosis [32]. However, the identification of *LZTR1* as a cause of schwannomatosis reduces the specificity of these more inclusive criteria and even the presence of bilateral VS is now no longer sufficient to be certain that an individual has NF2 [18, 20], although draft international consensus guidelines have retained bilateral VS as diagnostic for NF2. Furthermore, *LZTR1* germline pathogenic variants have been recently associated with higher risk of Unilateral Vestibular Schwannomas [19]. Therefore, the GG recommended that unilateral vestibular schwannomas should not be considered an exclusion criterion for the diagnosis of schwannomatosis in the absence of proven germline or mosaic NF2 [2, 25].

Segmental schwannomatosis is characterised by multiple schwannomas affecting one-limb or less than 5 contiguous segments of spine. The incidence of segmental forms among schwannomatosis patients remains to be determined precisely but has been reported as high as 30% in some series (27 out of 87 patients [33]). The genetics of segmental schwannomatosis remains incompletely understood with the description of germline *LZTR1* pathogenic variants in 33% [34] to 40% [35] of patients. Those findings suggest that segmental schwannomatosis might be different from a presumed somatic mosaicism.

Surgical resection of tumours seems to be effective on pain control in segmental schwannomatosis patients [34], but is characterised by a high rate of recurrence (5/9, 55% [34]), or by the systematic appearance of new tumours (4/4, 100% [36]). After surgery, neurological deficit seems to be more frequent than in sporadic cases, presumably due to the presence of several contiguous tumours in the same nerve, mimicking a rosary, but, in general, transient and clinical symptoms disappear in the month following surgery [36]. The GG recommendations reflect this.

Lastly, we have specifically included the psychological needs of patients who often have intractable pain that can hugely affect quality of life. Although schwannomatosis does not appear to affect life expectancy [2] it could be associated with an important emotional impact, including suicide, therefore the GG recommended assessment of psychological needs at annual visits.

There is clearly need for future research in schwannomatosis. A clear need is the development of better pain medication. The reason(s) why so few people who carry loss of function variants in *LZTR1* develop schwannomatosis is another important area for research as well as better prediction of MPNST risk and early detection. There is also a need to clarify the overlap with allelic conditions such as *LZTR1*-related Noonan syndrome as well as Coffin-Siris and rhabdoid tumour predisposition with *SMARCB1* variants. The latter also creates issues with incidental findings particularly in neonates or very young children [28, 37]. Genotype phenotype correlations are nonetheless strong with only minor overlap between rhabdoid predisposition and schwannomatosis with *SMARCB1* [37, 38].

In summary we have produced consensus recommendations for people affected or at risk of schwannomatosis that had high levels of agreement through four rounds of Delphi amongst a large peripatetic expert and patient group.

## Website

The complete guidelines can be downloaded from the ERN website: https://www.genturis.eu.

**Table 1:** Recommendations.

**Clinical overview recommendations**		**Strength**
Rec. 1	Life expectancy in schwannomatosis **is not** usually affected, unlike NF2. Pain **is** a prominent feature, especially for people with a *LZTR1* germline pathogenic variant.	Strong
Rec. 2	A changing tumour, in someone with *SMARCB1* germline pathogenic variant, especially one causing functional impairment, **should** prompt exclusion of malignant transformation.	Strong
Rec. 3	*LZTR1* germline pathogenic variant is associated with higher risk of unilateral vestibular schwannomas; therefore these tumours **should not** be considered an exclusion criterion for the diagnosis of schwannomatosis.	Strong
**Diagnosis recommendations**		**Strength**
Rec. 1	Germline pathogenic variant in *SMARCB1* or *LZTR1* **should** be considered diagnostic of schwannomatosis in the presence of someone with a proven schwannoma.	Strong
Rec. 2	Where possible, analysis of two tumours **should** be performed in sporadic cases to confirm or refute mosaic NF2. Schwannomatosis is characterised by multiple tumours harbouring independent somatic pathogenic variants in the *NF2* gene which are not present in their constitutional DNA.	Strong
Rec. 3	Baseline investigations to confirm schwannomatosis **should** include brain and internal auditory meati MRI with at least 3 mm and preferably ≤1 mm cuts through the internal auditory meatus to rule out bilateral vestibular schwannomas (NF2).	Moderate
Rec. 4	In people in whom schwannomatosis is clinically suspected and without germline pathogenic variants in *SMARCB1* or *LZTR1*, and without the diagnostic characteristics of NF2, RNA testing **should** be considered (for instance, for deep intronic *SMARCB1* variant associated with schwannomatosis). Due to the increased malignancy risk in schwannomatosis associated with *SMARCB1* this additional step is important as when found it allows confirmation of the diagnosis and the ability to offer pre-symptomatic testing to relatives.	Moderate
Rec. 5	In people with schwannomatosis at reproductive age or at transition, a discussion of the likely risks of transmission to offspring and the options for testing in pregnancy and pre-implantation diagnosis **should** be undertaken.	Strong
Rec. 6	Affected people and at-risk offspring **should** be told the risk of transmission is 50% in those with germline inherited variants. In those isolated cases with no family history with negative testing of *LZTR1* and *SMARCB1* the transmission rate is <10%. Reduced penetrance in *LZTR1* **should** be discussed.	Strong
**Imaging recommendations**		**Strength**
Rec. 1	For tumour surveillance or screening MRI **should** be used. PET scanning **should not** be used for diagnosis or surveillance of schwannomas.	Moderate
Rec. 2	A baseline assessment including full craniospinal MRI and/or whole-body MRI **should** be carried out as soon after diagnosis as the MRIs can be conducted without general anaesthetic (typically late childhood; 12–14 years) and should be repeated in early adulthood or if symptoms evolve.	Moderate
Rec. 3	The frequency of repeat MRI **should** be determined by clinical judgement guided by the presence of changing symptoms.	Moderate
Rec. 4	It is expected that routine repeat MRI are conducted at intervals of 2–3 years. More frequent MRI **should not** be conducted unless the person’s symptoms change.	Moderate
Rec. 5	In patients with localised pain and/or associated neurologic focal deficit, without an obvious schwannoma localised MRI **should** be performed using thin slices (<3 mm) in order to detect very small but functionally significant schwannomas.	Moderate
Rec. 6	For targeted investigation of pain, ultrasound (in the hands of someone experienced at imaging schwannomas) **may be** a useful problem-solving modality.	Weak
**Genotype specific imaging surveillance Recommendations** ***Please consider all recommendations in imaging recommendations****.*		**Strength**
Rec. 1	*SMARCB1*: the following baseline investigation **should** be performed at diagnosis: MRI brain and spine, and whole-body MRI.	Moderate
Rec. 2	*LZTR1:* the following baseline investigation **should** be performed at diagnosis: (1). High-resolution brain MRI with fine cuts (<3 mm) through the internal auditory canal and spine MRI (2). Whole body MRI. * *Note people with *LZTR1* pathogenic variants detected incidentally with no personal or family history of schwannomas and no pain or other schwannoma symptoms should not undergo MRI imaging to detect schwannomas as their risks are likely well below 1%.	Moderate
Rec. 3	If tumours are present at baseline MRI imaging, imaging **should** be repeated every 2–3 years, unless there is a change in symptoms or if tumours are present on brain imaging in which case an MRI at 12 months is indicated. Small (<1 cm) asymptomatic non-CNS tumours detected on whole body MRI particularly in the limbs **may not** require repeat imaging if no symptoms or signs develop.	Moderate
Rec. 4	If there is a change in symptoms, localised MRI **should** be performed according to clinical manifestations, and **should** be repeated at an increased frequency as determined by the clinical presentation.	Moderate
**Annual clinical assessment recommendation**		**Strength**
Rec. 1	At each review visit there **should** be: • Full assessment of pain history • Full neurological examination • Assessment of Quality of Life using a recognised tool e.g. EQ-5D • Assessment of psychological needs of the patient	Strong
**Non-surgical pain management recommendations**		**Strength**
Rec. 1	Multidisciplinary pain management focusing on symptom management and targeting pain related disability using a bio-psychosocial approach **should** be used.	Moderate
Rec. 2	Radiotherapy is likely to increase the risk of malignant transformation in people with schwannomatosis. Radiotherapy **should** only be considered in growing schwannomas that cannot be treated surgically or by other therapies.	Strong
Rec. 3	Painful schwannomas have a significant neuropathic component, drugs such as tricyclic antidepressants and gabapentinoids **should** be used first line, and SSRI or other ASD (Topiramate, Carbamazepine, Oxcarbazepine) second line.	Moderate
Rec. 4	Chronic use of opioids **is not** recommended due to their poor effect on neuropathic pain and associated tolerance, dependency and hyperalgesia.	Strong
Rec. 5	Transient Receptor Potential Vanilloid 1 (TRPV1) antagonists [capsaicin and some cannabinoid receptor ligands] **may be** effective in intractable pain because of Schwann cell expression of nerve growth factor.	Weak
**Surgical intervention recommendations**		**Strength**
Rec. 1	For those with painful schwannomas, if surgery is possible without neurological deficit, then early surgical intervention **should** be offered.	Strong
Rec. 2	If surgery is performed on symptomatic schwannomas, it **should** be by surgeons with experience resecting nerve sheath tumours.	Strong
Rec. 3	Some lesions are not surgically removable, and operations are linked to increased morbidity. So, assessment of the likelihood of success and the risks of neurological deficit **should** include assessment by a surgeon with significant experience resecting nerve sheath tumours	Strong
Rec. 4	The use of intraoperative neurophysiological monitoring **should** be considered and is essential for surgery on critical nerves.	Moderate
Rec. 5	If surgery fails to relieve local pain or symptoms, repeated surgeries to the same symptomatic area **should** be avoided as they offer diminishing benefit to pain control and may contribute to worsening of the schwannomatosis pain syndrome.	Moderate
Rec. 6	Use of spinal cord stimulation is an emerging therapeutic option and **should** be considered by multidisciplinary teams on an individual basis.	Weak
**Non-surgical intervention recommendation**		**Strength**
Rec. 1	Bevacizumab **probably should** be actively considered along with all other treatment options in the multidisciplinary team review, specifically in patients with multiple rapidly enlarging tumours, which are symptomatic in terms of pain and/or neurological deficit, and for those which are inoperable.	Weak

## Disclaimer

The content of these guidelines represents the views of the authors only and it is their sole responsibility; it cannot be considered to reflect the views of the European Commission and/or the Consumers, Health, Agriculture and Food Executive Agency (CHAFEA) or any other body of the European Union. The European Commission and the Agency do not accept any responsibility for use that may be made of the information it contains.
